# Influence of killer immunoglobulin-like receptors genes on the recurrence rate of ocular toxoplasmosis in Brazil

**DOI:** 10.1590/0074-02760220203

**Published:** 2023-03-31

**Authors:** Daiana de Souza Perce-da-Silva, Thays Euzebio Joaquim, Ana Luisa Quintella do Couto Aleixo, Juliana Pessanha Rodrigues Motta, Josué da Costa Lima-Junior, Marcelo Ribeiro-Alves, Joseli de Oliveira-Ferreira, Luís Cristóvão de Moraes Sobrino Porto, Dalma Maria Banic, Maria Regina Reis Amendoeira

**Affiliations:** 1 Fundação Oswaldo Cruz-Fiocruz, Instituto Oswaldo Cruz, Laboratório de Imunologia Clínica, Rio de Janeiro, RJ, Brasil; 2 Centro Universitário Arthur Sá Earp Neto, Faculdade de Medicina de Petrópolis, Laboratório de Imunologia Básica e Aplicada, Petrópolis, RJ, Brasil; 3 Fundação Oswaldo Cruz-Fiocruz, Instituto Oswaldo Cruz, Laboratório de Toxoplasmose e outras Protozooses, Rio de Janeiro, RJ, Brasil; 4 Fundação Oswaldo Cruz-Fiocruz, Instituto Nacional de Infectologia Evandro Chagas, Laboratório de Pesquisa Clínica em Oftalmologia Infecciosa, Rio de Janeiro, RJ, Brasil; 5 Universidade do Estado do Rio de Janeiro, Laboratório de Histocompatibilidade e Criopreservação, Rio de Janeiro, RJ, Brasil; 6 Fundação Oswaldo Cruz-Fiocruz, Instituto Oswaldo Cruz, Laboratório de Imunoparasitologia, Rio de Janeiro, RJ, Brasil; 7 Fundação Oswaldo Cruz-Fiocruz, Instituto Nacional de Infectologia Evandro Chagas, Centro de Pesquisa Clínica HIV/AIDS, Rio de Janeiro, RJ, Brasil

**Keywords:** ocular toxoplasmosis, KIR receptors, *Toxoplasma gondii* infection

## Abstract

**BACKGROUND:**

Recurrence is a hallmark of ocular toxoplasmosis (OT), and conditions that influence its occurrence remain a challenge. Natural killer cells (NK) are effectors cells whose primary is cytotoxic function against many parasites, including *Toxoplasma gondii*. Among the NK cell receptors, immunoglobulin-like receptors (KIR) deserve attention due to their high polymorphism.

**OBJECTIVES:**

This study aimed to analyse the influence of KIR gene polymorphism in the course of OT infection and its association with recurrences after an active episode.

**METHODS:**

Ninety-six patients from the Ophthalmologic Clinic of the National Institute of Infectology Evandro Chagas were followed for up to five years. After DNA extraction, genotyping of the patients was performed by polymerase chain reaction sequence-specific oligonucleotide (PCR-SSO) utilising Luminex equipment for reading. During follow-up, 60.4% had a recurrence.

**FINDINGS:**

We identified 25 KIR genotypes and found a higher frequency of genotype 1 (31.7%) with worldwide distribution. We note that the *KIR2DL2* inhibitor gene and the gene activator *KIR2DS2* were more frequent in patients without recurrence. Additionally, we observed that individuals who carry these genes progressed recurrence episodes slowly compared to individuals who do not carry these genes.

**MAIN CONCLUSIONS:**

The KIR2DL2 and KIR2DS2 are associated as possible protection markers against ocular toxoplasmosis recurrence (OTR).

*Toxoplasma gondii* is an obligate intracellular protozoan parasite that belongs to the phylum apicomplexa, subclass coccidia. The parasite has a worldwide distribution with a high prevalence that infects humans, birds, rodents, and other animals (intermediate hosts) and felids (definitive hosts) on all continents. Toxoplasmosis can be classified as congenital or acquired. Congenital toxoplasmosis may be exclusively ocular or accompanied by systemic or central nervous system changes.^1^

In recent decades, advances have shown that toxoplasmosis is one of the most important causes of posterior uveitis globally, representing up to 85% of all cases.^1^ ^,^ ^2^ ^,^ ^3^ The ocular lesions are characterised by necrotising retinitis with oval or circular lesions. Besides it, the lesion can remain active for weeks, and even after healing, it may contain *T. gondii* cysts, so the protozoan remains viable in tissues for years.^4^

Ocular toxoplasmosis (OT) is a disease characterised by recurrence episodes. However, the conditions associated with recurrence episodes have not been completely elucidated. After infection, the ocular symptoms depend on complex and variable factors, such as socioeconomic factors and the parasite genotype.^2^

The parasite contrives to manipulate the immune response in the eyes favouring its survival without causing too much damage to the organ.^5^ In the early phase of *T. gondii* infection, innate immunity cells are recruited to the site of infection. Natural killer (NK) are important lymphocytes acting in the acute phase of toxoplasmosis.^6^ *In vivo* studies in mice revealed that controlling *T. gondii* requires the early production of the pro-inflammatory cytokine IL-12, which stimulates NK, CD4^+^, and CD8^+^ lymphocytes to release IFN-γ.^6^ ^,^ ^7^ ^,^ ^8^ Studies recent in murine and human models revealed the existence of noncirculating NK cells that remain resident in the peripheral tissues, termed tissue-resident NK (trNK).^9^ Resident and recruited innate and adaptive immune cells maintained at the ocular surface.^10^

Control of NK cell action is through membrane receptors, including killer immunoglobulin-like receptors (KIR), which recognise human leukocyte antigen class I molecules (HLA class I) expressed by most cells in the body. Similar to toll-like receptors (TLR) and others present in innate immune cells, the KIR genetic diversity is determined through the expression of multiple genetically encoded receptors. Thus, expression is essentially at random during development and may express multiple KIR.^11^ The extensive genetic polymorphism of KIR receptors and the regulation of their expression in different NK cell clones are essential factors that delineate each individual’s innate and adaptive immune response.

NK cells have great importance in controlling *T. gondii* infection; however, the role of *KIR* genes that encode the immune receptors of NK cells and can trigger local inflammation in the eye has not been elucidated in ocular toxoplasmosis yet. *KIR* genes have been described as risk or protective factors in different inflammatory ocular diseases^12^ ^,^ ^13^ ^,^ ^14^ and are also associated with many other infectious diseases.^15^ ^-^ ^22^

To date, KIR receptors with ocular toxoplasmosis involving recurrence events have been examined in only one study.^23^ Some studies suggest that the development of ocular lesions is a result of host genetic susceptibility and exposure to virulent strains.^24^ ^,^ ^25^

Thus, the characterisation of these receptors in individuals with ocular toxoplasmosis may help to understand their role in regulating the immune response, clinical evolution of the disease, and their relationship with faster or lower recurrences. Besides it, the identification of predisposal individuals may help in their clinical management. However, histological analyses of the ocular tissue affected by *T. gondii* and NK cytotoxicity assays should be conducted to better understand the role of NK cells and the expression of KIR in the immunopathogenesis of ocular toxoplasmosis.

## MATERIALS AND METHODS

*Ethics statement* - The Research Ethics Committee of the National Institute of Infectology Evandro Chagas (INI/Fiocruz) approved this study protocol as a subproject under the CAAE 0075.0.009.000-11. After being informed about the study’s nature, all the volunteers gave written informed consent, including the objectives and laboratory procedures performed. They allowed the store and future use in the research of their samples.

*Patients* - This study was carried out with 96 blood and serum samples stored in the Toxoplasmosis Laboratory of the IOC-Fiocruz. The patients included in this study were part of previous works by our group that had a larger sample size of 274 patients.^26^ ^,^ ^27^ The patients were attended by the same ophthalmologist between January 2010 and January 2014, and follow-up until July 2015 at the outpatient unit of the Infectious Ophthalmology Laboratory of the National Institute of Infectology Evandro Chagas at Fiocruz.^26^

For this study, the patients were classified according to the recurrence of ocular toxoplasmosis. As previously described in Aleixo et al.,^26^ ^,^ ^27^ recurrent cases were defined as active retinochoroiditis associated with a retinal scar in either eye.^28^ It’s important to highlight that episodes of inflammation of the anterior segment in eyes with retinochoroiditis scars were not considered a recurrence.^29^ Creamy-white focal retinochoroidal lesions in the absence of other retinochoroidal scars were considered primary lesions. Primary retinochoroidal lesion cases with no recurrence were supposed to be highly probable of ocular toxoplasmosis and thus included in the follow-up.^27^ The follow-up criteria adopted to involve patients in the non-recurrence group are described in more detail in Aleixo et al.^26^ It has been demonstrated that the risk of OT recurrence is higher in the year following the first infection than in future years.^26^ ^,^ ^30^ However, to avoid erroneous associations, we included in the non-recurrence group only patients who were followed up for at least two years.

Patients’ exclusion criteria were pregnant during any recurrent episodes, genetically related, having comorbidities (*e.g.*, chronic renal failure), systemic infections (*e.g.*, acquired immunodeficiency syndrome (AIDS), syphilis, and tuberculosis), autoimmune diseases, history of intravenous drugs use, single and unilateral or multiple exudative lesions of retinochoroiditis, and history of cancer chemotherapy or immunosuppressive drug or peri- and/or intraocular steroids use. All exams were performed in the Laboratory of Immunology and Immunogenetics (INI/Fiocruz).

*Genomic DNA extraction* - According to the manufacturer’s instructions, genetic DNA was isolated from peripheral blood samples collected in EDTA using a QIAamp^®^ DNA Blood Midi/Maxi Kit (Qiagen, Valencia, CA). The DNA concentration was determined using a Qubit fluorimeter (Life Technologies, Carlsbad, CA), and the filtrates containing the isolated DNA were stored at -20ºC until the time of use.

*KIR genotyping* - The reverse sequence-specific oligonucleotide technique (One Lambda Inc., Canoga Park, CA) with Luminex xMap technology (Luminex Corp., Austin, TX) was used for the typing of the 14 KIR genes and two KIR pseudogenes, namely *KIR2DL1, KIR2DL2, KIR2DL3, KIR2DL4, KIR2DL5, KIR2DS1, KIR2DS2, KIR2DS3, KIR2DS4, KIR2DS5, KIR3DL1, KIR3DL2, KIR3DL3, KIR3DS1, KIR2DP1,* and KIR3DP1, according to the manufacturer’s instructions. The carrier frequency was calculated by directly counting individuals owning at least one copy of the gene. The genotypic frequency (GF) was calculated by the formula GF = 1-√(1-CF), where CF indicates the carrier frequency, calculated by CF = frequency(F)%/100.^31^ The survey data were recorded and entered into an Epi Info 2007 (Centres for Disease Control and Prevention, Atlanta, GA) database.

*Statistical analysis* - Student’s T-tests were used to compare clinical-demographic features for continuous numerical variables between recurrent and non-recurrent groups. Shapiro-Wilk Normality Tests were used to confirm the normality of the variable samples. In contrast, Chi-square tests were used to evaluate frequency independence among features and the groups for categorical nominal variables. To analyse recurrent event data, where patients experienced the event of interest multiple times throughout the follow-up period, ocular toxoplasmosis recurrence (OTR), we used Mixed-effects Cox Proportional Hazard Models. The inherent correlation within patients was accounted for, including a frailty term. The absence of recurrence has defined censoring until the date of the last visit. The effects of potential Hazard factors on OTR were assessed using Hazard ratios (HR) and their confidence interval (CI) 95%. Confounding variables were selected by Simple/Bivariate models and included in multivariate models if any p-value < 0.2 to eliminate sampling bias. For each participant, person-years (pY) at risk were calculated between the discharge date of a previous episode and the discharge date of the last episode of ocular toxoplasmosis or the last follow-up visit, which occurred first. Crude incidence of OTR per 100 py, *i.e.*, the number of OTR episodes divided by pY and multiplied by 100, and its CI 95%, estimated according to asymptotic standard errors calculated from a Gamma distribution.^32^ All statistical analyses were performed using R version 3.6.1^33^ and packages ‘coxme’ version 2.2-16 and ‘jstable’ version 1.0.7. Code will be made available by the authors after reasonable requests.

## RESULTS

*General features of the studied population* - The clinical-demographic data summarised in Table I show that patients with ocular toxoplasmosis were followed up for different periods. There was a homogeneous sex distribution in the study population, with most of the individuals being adults (31 years old on average) and residents of the state of Rio de Janeiro (97/90.6%).

**TABLE I:** Clinical-demographic features of patients consulted at the outpatient unit of the Infectious Ophthalmology Laboratory of the National Institute of Infectology Evandro Chagas, Fiocruz, RJ (N = 96)

Features	Levels	Overall	Recurrences		p
			No	Yes	
Gender	M	54 (56.2%)	20 (52.6%)	34 (58.6%)	0.7
	F	42 (43.8%)	18 (47.4%)	24 (41.4%)	
State	Rio de Janeiro	87 (90.6%)	35 (92.1%)	52 (89.7%)	0.6
	Others*	9 (9%)	3 (7%)	6 (10,3%)	
Age**		14.18-66.98	14.18-55.85	15.87-66.98	0.1
Follow-up in days (Mean (SD))		1338.53 (327.43)	1261.82 (321.37)	1388.79 (324.27)	0.06

SD: standard deviation; *Alagoas, Espírito Santo, Mato Grosso, Minas Gerais, Paraíba, and Rio Grande do Sul; **min-max; Student’s T-tests were used for continuous numerical variables and Chi-square tests were used for categorical nominal variables.

The individuals were followed for different periods, and the majority (92/96%) had a follow-up of more than two years. We observed that 46 individuals (47.9%) were followed up for more than 4 years (Table II). As for the number of recurrences, there was a variation between one and five episodes, 33 individuals (34.3%) had one relapse episode, 18 (18.7%) had two episodes, five (5.2%) had three episodes and two (3.1%) had five relapse episodes during the follow-up period (Table II).

**TABLE II:** Follow-up time and number of recurrence of patients consulted at the outpatient unit of the Infectious Ophthalmology Laboratory of the National Institute of Infectology Evandro Chagas, Fiocruz, RJ (N = 96)

		Follow up				
		> 1 and ≤ 2 years (N = 4)	> 2 and ≤ 3 years (N = 18)	> 3 and ≤ 4 years (N = 28)	> 4 and ≤ 5 years (N = 45)	> 5 and ≤ 6 years (N = 1)
N recurrence	0		12	12	14	
	1	4	4	11	14	
	2		2	4	12	
	3			1	3	1
	5				2	
	Total	4	18	28	45	1

*KIR genes frequencies* - The distribution of KIR gene frequencies (*F*%) in patients consulted at the outpatient unit of the Infectious Ophthalmology Laboratory from INI is illustrated in Table III. All 16 KIR genes investigated (14 genes and two pseudogenes, including framework loci) were detected in the study population, and the framework loci *KIR2DL4*, *KIR3DL2*, *KIR3DL3*, and *KIR3DP1* were present in all individuals. In general, the frequencies of inhibitory KIR genes were higher than 90%, except for the *KIR2DL2* and *KIR2DL5* genes (54.2% and 54.5%, respectively). Also, the proportion of patients carrying any activating KIR gene ranged from 18.7% to 51%, except for the KIR2DS4, with a 96.9% frequency.

**TABLE III:** Distribution of NK cell receptors, immunoglobulin-like receptors (KIR) gene frequencies in patients of the Infectious Ophthalmology Clinic of the National Institute of Infectology Evandro Chagas, Fiocruz, RJ

*KIR* gene	N	F%	GF
Inhibitory			
*2DL1*	94	97.9	0.856
*2DL2*	52	54.2	0.324
*2DL3*	88	91.7	0.712
*2DL5*	53	55.2	0.331
*3DL1*	95	98.9	0.896
Activating			
*2DS1*	40	41.7	0.237
*2DS2*	49	51	0.3
*2DS3*	18	18.7	0.099
*2DS4*	93	96.9	0.824
*2DS5*	41	42.7	0.244
*3DS1*	34	35.4	0.197
Framework			
*2DL4*	96	100	1
*3DL2*	96	100	1
*3DL3*	96	100	1
^*A*^ *3DP1*	96	100	1
Pseudogene			
*2DP1*	93	96.9	0.824

N: number of individuals; F%: KIR genes frequencies; GF: genotypic frequency; A: framework, and pseudogene.

*KIR genotypes* - A total of 24 different KIR genotypes were identified in the studied population (Figure) based on the presence and absence of 16 KIR genes. These were previously described in the KIR genotype database (www.allelefrequencies.net) and contained between eight and 16 KIR genes per individual. Genotype 1, the most common, accounted for 31.3% of all genotypes in our studied population, and genotypes 4, 2, and 3 were detected in 12.5%, 11.5%, and 8.3% of the studied population, respectively (Figure). Among the 24 genotypes, more than half (14 genotypes) were in low frequency, presenting only 1% of the population. The two most common genotypes (1 and 4) observed in our population were present at a similar frequency to that of North, Central, and South American populations (www.allelefrequencies.net). The population was classified according to the haplogroups (A and B) existing for the KIR genes. Haplogroup B was named Bx because of the lack of distinction between AB and BB’s haplotype. The haplogroup Bx was the most frequent (68.7%), while the haplogroup AA was present in 31.3% of our population.

**Figure:**
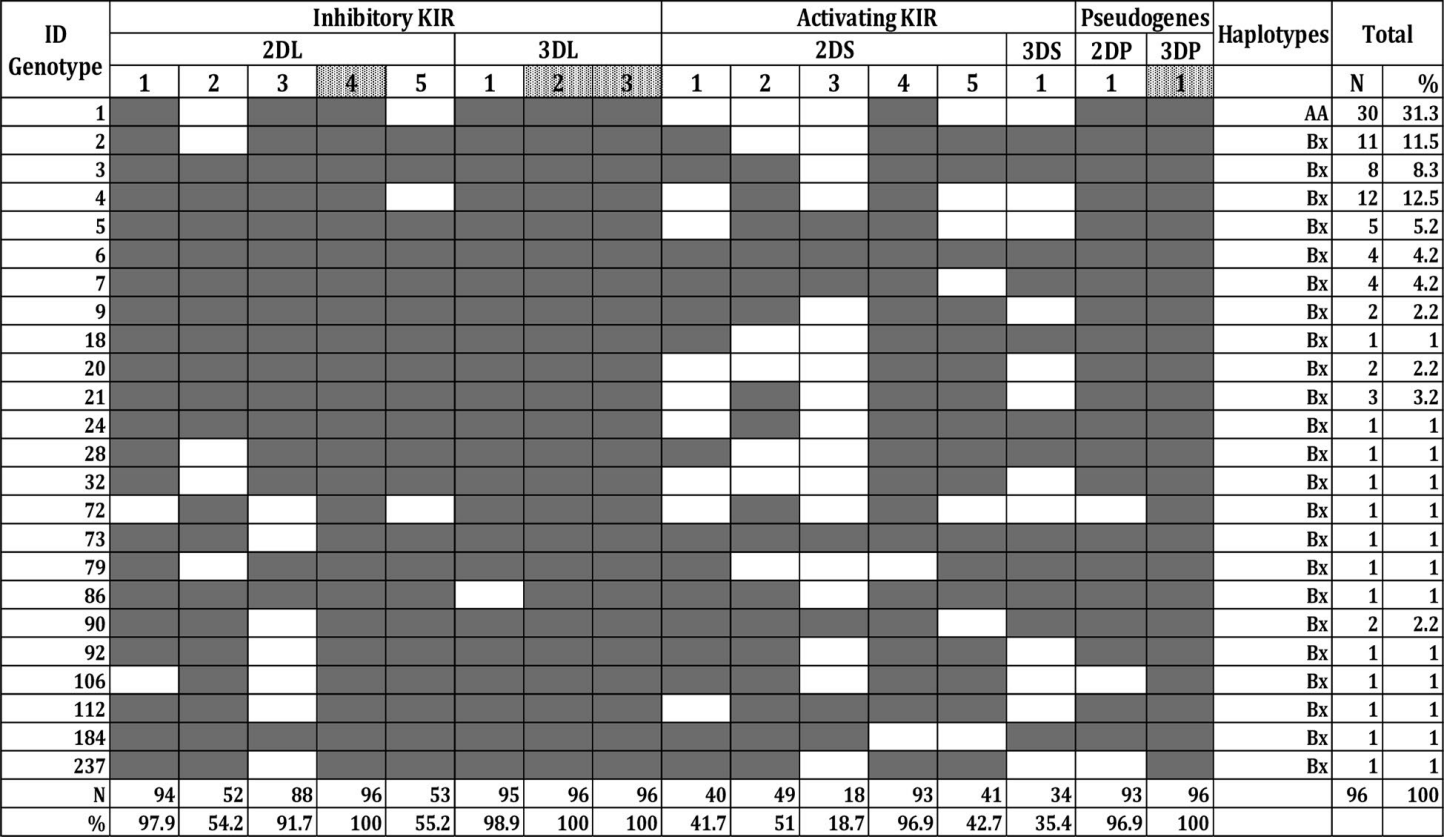
NK cell receptors, immunoglobulin-like receptors (KIR) genotype profiles in the study population. ID: identification number in allefrequencies.net; The filled boxes indicate the presence of the gene; white boxes indicate the absence of the gene; the dot boxes represent the framework genes.

When we evaluated a possible association between KIR genes and the progression for recurrence of ocular toxoplasmosis after an active episode, we used Mixed-effects Cox Proportional Hazard Models considering all 96 patients and their recurrences (94 events). The inherent correlation within patients was accounted for, including a frailty term. We observed that individuals with KIR2DL2 (aHR: 0.63, p = 0.036) and KIR2DS2 (aHR: 0.63, p = 0.045) genes progressed recurrence episodes slowly compared with individuals without these genes (Table IV).

**TABLE IV:** Simple and multiple mixed-effects cox proportional Hazard models for the time until each recurrence in 96 patients (94 events) from an outpatient unit of the Infectious Ophthalmology Laboratory of the National Institute of Infectology Evandro Chagas, Fiocruz, RJ

Features	Levels	Outcome	pY	Crude incidence by 100 pY (CI 95%)	HR (CI 95%)	aHR (CI 95%)	p
Overall		94	268.410	0.35 (0.28-0.43)			
KIR2DL1	0	1	5.27	0.19 (0-1.06)	Reference	Reference	Reference
	1	93	263.14	0.35 (0.29-0.43)	2.32 (0.18,30.61)	1.33 (0.18,9.67)	0.781
KIR2DL2	0	55	116.43	0.47 (0.36-0.61)	Reference	Reference	Reference
	1	39	151.98	0.26 (0.18-0.35)	0.47 (0.25,0.87)	0.63 (0.41,0.97)	0.036
KIR2DL3	0	5	16.41	0.3 (0.1-0.71)	Reference	Reference	Reference
	1	89	252	0.35 (0.28-0.43)	1.16 (0.34,4)	0.84 (0.33,2.11)	0.707
KIR2DL5	0	38	112.11	0.34 (0.24-0.47)	Reference	Reference	Reference
	1	56	156.3	0.36 (0.27-0.47)	0.87 (0.45,1.68)	0.95 (0.62,1.45)	0.807
KIR2DP1	0	2	6.78	0.29 (0.04-1.07)	Reference	Reference	Reference
	1	92	261.63	0.35 (0.28-0.43)	1.27 (0.18,8.81)	0.87 (0.21,3.58)	0.846
KIR2DS1	0	49	155	0.32 (0.23-0.42)	Reference	Reference	Reference
	1	45	113.42	0.4 (0.29-0.53)	1.13 (0.6,2.15)	1.21 (0.8,1.82)	0.361
KIR2DS2	0	59	124.49	0.47 (0.36-0.61)	Reference	Reference	Reference
	1	35	143.92	0.24 (0.17-0.34)	0.44 (0.24,0.82)	0.63 (0.4,0.99)	0.045
KIR2DS3	0	80	226.19	0.35 (0.28-0.44)	Reference	Reference	Reference
	1	14	42.22	0.33 (0.18-0.56)	1.09 (0.47,2.53)	1.12 (0.63,2)	0.705
KIR2DS4	0	1	10.89	0.09 (0-0.51)	Reference	Reference	Reference
	1	93	257.52	0.36 (0.29-0.44)	5.09 (0.46,55.88)	2.91 (0.4,21.04)	0.291
KIR2DS5	0	48	142.17	0.34 (0.25-0.45)	Reference	Reference	Reference
	1	46	126.24	0.36 (0.27-0.49)	0.84 (0.44,1.61)	0.92 (0.61,1.41)	0.707
KIR3DS1	0	56	170.63	0.33 (0.25-0.43)	Reference	Reference	Reference
	1	38	97.78	0.39 (0.28-0.53)	1.09 (0.56,2.11)	1.09 (0.72,1.65)	0.694

Levels 0 and 1 indicate non-carriers and carriers, respectively of the corresponding NK cell receptors, immunoglobulin-like receptors (KIR) genes indicated on the left; outcome: number of ocular toxoplasmosis (OTR) episodes; pY = person years of follow-up; crude incidence by 100 pY = number of OTR episodes divided by pY and multiplied by 100 and its 95% confidence interval; HR: Hazard-ratios estimated after mixed-effect cox proportional Hazard models; aHR: adjusted Hazard-ratios by age and the number of OTR confounders; CI: confidence interval.

## DISCUSSION

Because of KIR’s role in the immune response and its extensive genomic diversity, it is known that variation in KIR genes affects the resistance and susceptibility to the pathogenesis of various diseases.^34^ In this context, the current study characterised the gene frequency profile of the 16 genes encoding KIR receptors and evaluated their influence in patients with recurrence and non-recurrence ocular toxoplasmosis after active episodes.

Concerning the profile of the gene frequencies of KIR receptors in the study population, we observed that the genes KIR3DL1, KIR2DL1, KIR2DS4, and KIR2DP1 were the most prevalent (above 97%). These high frequencies are similar to those in other Brazilian populations, including Rio de Janeiro.^22^ ^,^ ^31^ ^,^ ^35^ ^-^ ^39^

NK cells are fast-acting innate immune cells that provide a first line of defence of the immune system by killing direct the microorganism and/or the infected cells and producing pro-inflammatory cytokines. And indirectly, NK cells regulate adaptive immunity via crosstalk with dendritic cells and by the production of chemokines and cytokines.^40^ ^,^ ^41^

NK cells present important functions in different phases of the immune response against parasite infection. Firstly, during the innate response, *T. gondii* infection triggers the production of inflammatory cytokines IL-1β, IFNα/β, IL-6, IL-12, IL-15, and IL-18, driving NK cell production of IFNγ, resulting in early control of parasite infection by targeting intracellular parasites. Moreover, despite, the importance of NK cell IL-17 is not well understood, IL-6 can stimulate the production of IL-17 by activated NK cells. Even in the innate immune response phase, CD8 response induced by NK cells is also present. After this first activation moment, NK cells, which also produce IL-10, can regulate innate responses by down-regulating IL-12 and possibly other cytokines. Whether NK cell IL-10 can impact CD4 and CD8 T cell responses remains unknown, but recent evidence describes its potential in the regulation of innate immune response against *T. gondii* ^42^ and NK cells negatively regulate CD8 T cells to promote immune exhaustion and chronic *T. gondii* infection.^43^ Lastly, the concept that NK cells are only innate immune cells is changing. Evidence supports their development of memory-like traits. Human studies have identified NKG2C+ NK cells to have memory-like traits.^42^ NK cells can participate in adaptive immunity as memory-like cells.^44^ Therefore, in *T. gondii*, it may be important for secondary infections. Whether NK cells that experience *T. gondii* infection early live long-term or develop memory-like features and the mechanisms behind these cell-intrinsic fates are still under investigation, but KIR receptors probably participate in this process.

*Toxoplasma gondii* infection have multiple and complex roles at all phases of immunity to this parasite.^42^ Furthermore, the cytokines and other soluble factors can be modulating the activity of NK cells by the interaction of surface receptors with their respective ligands, including KIR receptors and their HLA ligands.^34^

In the context of infectious disease, KIR molecules play an important role in aiding the immune response and have been shown a different effect in a variety of settings.^45^ The current study found that the inhibitory gene KIR2DL2 and the activator KIR2DS2 act as protective markers. It is because both genes are more frequent in the non-recurrence group. In addition, the individuals with these genes have a longer time between the recurrence episodes. The following hypothesis may explain this protective effect. Although NK cells are known to release interferon-gamma (IFN-γ) within hours after infection with *T. gondii*, these cells are very important in chronic inflammation.^46^ ^,^ ^47^ These genes modulate NK cells’ killer function, and the possible expression inhibiting signals may decrease inflammatory response and slow the recurrence of ocular toxoplasmosis. Therefore, future studies should address the functional characterisation of these genes and their respective HLA ligands.

The KIR2DL2 and KIR2DS2 genes are in strong linkage disequilibrium in most populations worldwide.^48^ ^,^ ^49^ ^,^ ^50^ ^,^ ^51^ ^,^ ^52^ Due to this strong linkage disequilibrium between the two loci, it is difficult to separate the effect of one locus from the other. That is, it is difficult to determine which one is mediating the effect.^53^ In our population, the presence of these genes is, for the most part, in coexistence. Of the 52 individuals who have the KIR2DL2 gene, 49 also have the KIR2DS2.

The inhibitor gene KIR2DL2 and its corresponding activator KIR2DS2, have already been associated with other diseases. They have been associated with protection against HIV-1 infection,^54^ KIR2DL2+HLA-C1 was associated with a decreased risk of chronic HBV^16^ and the combination of KIR2DS2+HLA-C1 was associated with a protective effect against adverse outcomes of coronavirus disease (COVID-19) in Sardinia, an Italian island in the Mediterranean Sea.^55^ In contrast, a previous study that determined and compared the frequencies of the KIR genes of children with severe or uncomplicated malaria with healthy controls in the same area found that the frequencies of both genes were significantly higher in malaria cases (severe or uncomplicated) than in controls. The authors conclude that KIR2DL2 + C1 and/or KIR2DS2 + C1 carriers were more at risk of being infected with malaria parasites than those without any of these genotypes.^53^ Interestingly, Seich et. al.^41^ reported a consistent effect of KIR2DL2 enhances HLA class 1- restricted CD8+ T cell-mediated adaptive immunity. These authors postulated that, in the presence of chronic infection, protective T cells survive longer if they carry KIR2DL2 and therefore exert stronger protection.^41^ These results suggest that KIRs play a role in the occurrence and persistence of infectious diseases.

In the present study, we characterise the KIR genes’ profile in patients with ocular toxoplasmosis after an active episode. We observed two genes (KIR2DL2 and KIR2DS2) acting together as possible protection markers against OTR. The current study is primarily focused on gene frequency and disease. In this context, our study has some limitations, such as we did not perform experiments to test HLA ligand genes, as well as the phenotype of NK cells was also not analysed, however in this study, the main focus was to evaluate the influence of the polymorphism KIR gene on the course of infection of OTR and its association with recurrences after an active episode. In addition, we plan to extend our investigations to analyse the impact of KIR and its combinations of HLA ligands and high-resolution KIR genotyping concerning alleles with high and low expression on NK cells.

As far as we know, no literature relates to the polymorphism of KIR and the follow-up of recurrence of ocular toxoplasmosis for a prolonged period (up to 5 years). Thus, the characterisation of KIR genes makes this study a pioneer in searching for an association between the KIR genes polymorphisms and the time until the recurrence of ocular toxoplasmosis.
